# *Giardia duodenalis* extracellular vesicles regulate the proinflammatory immune response in mouse macrophages* in vitro* via the MAPK, AKT and NF-κB pathways

**DOI:** 10.1186/s13071-021-04865-5

**Published:** 2021-07-08

**Authors:** Panpan Zhao, Lili Cao, Xiaocen Wang, Jianhua Li, Jingquan Dong, Nan Zhang, Xin Li, Shan Li, Min Sun, Xichen Zhang, Min Liang, Xudong Pu, Pengtao Gong

**Affiliations:** 1 Key Laboratory of Zoonosis, College of Veterinary Medicine, Jilin University, Changchun, 130062 People’s Republic of China; 2 Jilin Academy of Animal Husbandry and Veterinary Medicine, Changchun, 130062 People’s Republic of China; 3 Jiangsu Key Laboratory of Marine Biological Resources and Environment, Jiangsu Key Laboratory of Marine Pharmaceutical Compound Screening, Co-Innovation Center of Jiangsu Marine Bio-Industry Technology, Jiangsu Ocean University, Lianyungang, 222005 People’s Republic of China

**Keywords:** *Giardia duodenalis*, Extracellular vesicles, Immune response, MAPK

## Abstract

**Background:**

*Giardia duodenalis* is an extracellular protozoan parasite that causes giardiasis in mammals. The presentation of giardiasis ranges from asymptomatic to severe diarrhea, and the World Health Organization lists it in the Neglected Diseases Initiative. Extracellular vesicles (EVs) are a key mediator of intracellular communication. Although previous studies have shown that *G. intestinalis* can regulate a host’s innate immune response, the role of *G. intestinalis* EVs (GEVs) in triggering a *G. intestinalis-*induced innate immune response remains to be further explored.

**Methods:**

In this study, GEVs, *G. intestinalis* and GEVs + *G. intestinalis* were inoculated into macrophages, respectively. The transcription and secretion levels of proinflammatory cytokines, including interleukin (IL)-1β, IL-6 and tumor necrosis factor alpha (TNF-α), were measured using real-time quantitative PCR (qPCR) and enzyme-linked immunosorbent assays (ELISAs). The phosphorylation levels of the MAPK, AKT and NF-κB signaling pathways in GEV-stimulated mouse macrophages were examined using western blotting and immunofluorescence methods. The roles of activated pathways in the GEV-triggered inflammatory response were determined using inhibition assays, western blotting and ELISAs.

**Results:**

The results showed that pretreatment with GEVs enhanced with *G. intestinalis* (GEVs + *G. intestinalis*) induced IL-1β, IL-6 and TNF-α transcription and secretion from mouse macrophages compared to stimulation with either GEVs or *G. intestinalis* alone. Inoculation of mouse macrophages with GEVs upregulated the phosphorylation levels of the p38 MAPK, p44/42 MAPK (Erk1/2), AKT and NF-κB signaling pathways and led to the nuclear translocation of NF-κB p65. Blocking the activated p38, Erk and NF-κB signaling pathways significantly downregulated the secretion of proinflammatory cytokines, and blocking the activated AKT signaling pathway demonstrated reverse effects.

**Conclusions:**

The results of this study reveal that GEVs can enhance *G. intestinalis-*induced inflammatory response levels in mouse macrophages through activation of the p38, ERK and NF-κB signaling pathways. The role of GEVs in regulating host cell immune responses may provide insights into exploring the underlying mechanisms in *G. intestinalis*–host interactions.

**Graphical abstract:**

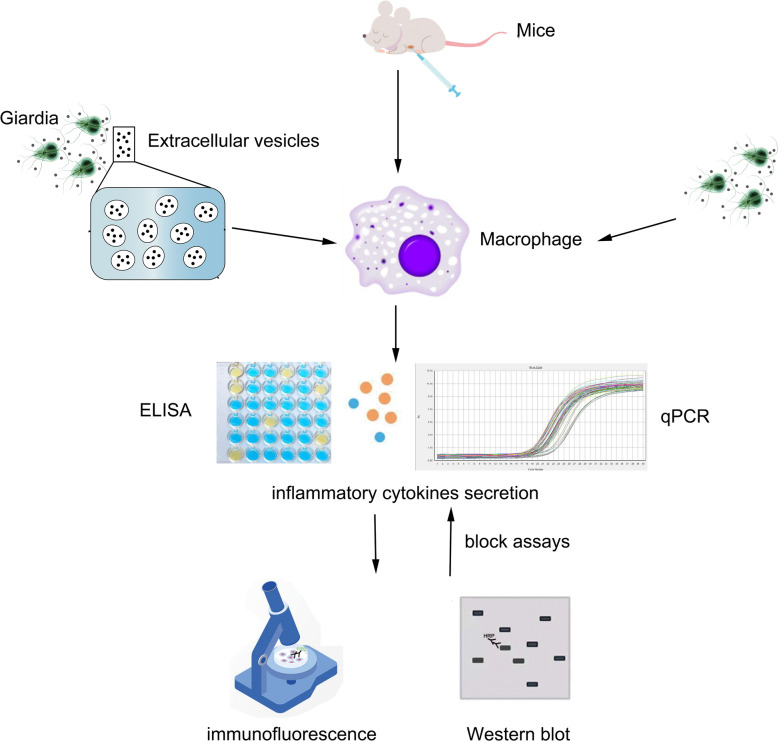

**Supplementary Information:**

The online version contains supplementary material available at 10.1186/s13071-021-04865-5.

## Background

*Giardia duodenalis* is a protozoan parasite that colonizes the small intestine [1]. The ingestion of water or food contaminated with *G. intestinalis* cysts by susceptible mammalian hosts leads to giardiasis, especially in young children, with clinical presentations that range from no symptoms at all to severe diarrhea [2, 3]. There are an estimated 280 million cases of diarrhea due to assemblages A and B of *G. intestinalis*-induced giardiasis annually [4]. Giardiasis has drawn public attention, and the World Health Organization lists it in the Neglected Diseases Initiative [5]. Innate immunity represents the first line of defense against pathogen invasion through the recognition of a vast array of pathogen-associated molecular patterns (PAMPs). A better understanding of vital PAMPs from *G. intestinalis* and exploration of its roles in the immune mechanisms of host resistance to infection with *G. intestinalis* will provide new insights into effective strategies against giardiasis.

Intracellular communication occurs via the delivery of biological molecules, including proteins, lipids and nucleic acids, by extracellular vesicles (EVs) [6]. EVs range in size from 30 to 1000 nm in diameter and are classified into microvesicles, exosomes, ectosomes and apoptotic bodies [7, 8]. Most cells, including immune cells, tumor cells and blood cells, secrete EVs that bind to pattern recognition receptors on target cells, and EVs may be used to deliver biological molecules into cells to activate intracellular signaling pathways and trigger immune responses [9, 10]. Many parasites, including *Trichomonas vaginalis*, *Trypanosoma brucei*, *Ascaris suum*, *Leishmania* and *Echinococcus multilocularis*, secrete EVs to modulate host–pathogen communication [11–15]. *Giardia intestinalis* is an extracellular pathogen that adheres to the surface of enterocytes but does not enter these cells [1]. It releases various kinds of EVs, which are captured by human immature dendritic cells, and these EVs are also involved in *G. duodenalis* adhesion to enterocytes [16–18].

Macrophages are the most important and powerful phagocytic cells and are widely distributed in the tissues of mammals. These cells play roles in the elimination of invading pathogens [19]. Our previous study showed that mouse macrophages internalized *G. duodenalis* EVs (GEVs), which subsequently regulated the secretion of inflammatory cytokines [20]. Mitogen-activated protein kinase (MAPK) signaling pathways consist of several kinases, including p38 MAPK, extracellular signal-related kinase (ERK) and c-Jun-activated kinase (JNK). These pathways activate an orchestrated cascade, phosphorylate MAPK kinases, regulate cell proliferation and differentiation and control cell responses to cytokines and stress [21–23]. The nuclear transcription factors NF-kappaB (NF-κB) or Rel proteins regulate the expression of sets of genes and may influence a variety of biological processes, such as innate immunity, adaptive immunity, inflammation and stress responses [24]. Insulin and a variety of growth and survival factors activate the PI3K/Akt signaling pathway, which plays a critical role in controlling survival and apoptosis [25, 26]. The interaction of immune response mechanisms between GEVs and macrophages requires further elucidation.

In study reported here we enriched GEVs and then examined the roles of these vesicles in the *G. duodenalis*-induced inflammatory response, detected the protein expression levels of the MAPK, AKT and NF-κB signaling pathways and then determined the roles of these activated pathways in GEV-induced inflammatory responses in mouse macrophages, with the ultimate aim to determine the role of GEV regulation in inflammatory immune response mechanisms.

## Methods

### Animals

The Animal Welfare and Research Ethics Committee of Jilin University approved all of the mouse experiments (IACUC Permit Number: pzpx20190929065). Female C57BL/6 mice (6–8 weeks) were purchased from the Changsheng Experimental Animal Center (Anshan, China), fed sterilized food and water* ad libitum* and housed in feeding cages kept under a 12/12-h light/dark cycle.

### Parasites and GEVs

*Giardia duodenalis* trophozoites (WB strain clone C6, ATCC30957; American Type Culture Collection, Manassas, VA, USA) were grown for 48–72 h in modified TYI-S-33 medium with 12.5% fetal bovine serum (FBS; Every Green, Zhejiang, China) and 0.1% bovine bile (Sigma-Aldrich, Merck KGaA, Burlington, MA, USA) at 37 °C under microaerophilic conditions. Confluent trophozoites were passaged by placing the tubes on ice for 20 min and rubbing the tubes 50–100 times at 10-min intervals. The suspended trophozoites were centrifuged at 2000×*g* for 8 min and resuspended in fresh medium at a final concentration of 1 × 10^8^ parasites/ml.

GEVs were prepared as previously described with slight modifications [16–18, 27]. Specifically, the *G. duodenalis* trophozoites were diluted to 1 × 10^6^ parasites/ml and cultured for 12 h in modified TYI-S-33 medium with 12.5% exosome depleted of fetal bovine serum (Biological Industries, Beit HaEmek, Israel). GEVs in culture supernatants were purified through gradient centrifugation at 2000×*g* for 10 min and at 10,000×*g* for 45 min followed by ultracentrifugation at 100,000×*g* for 60 min after first being filtered through a 0.22-µm sterilized PES membrane (Merck Millipore, MilliporeSigma, Burlington, MA, USA). The pellets were then resuspended in 200 μl of sterilized phosphate-buffered saline (PBS) after two washes. The protein concentrations were quantified using a BCA protein assay kit (Thermo Fisher Scientific, Waltham, MA, USA).

### Preparation of mouse peritoneal macrophages

After an acclimatization period of > 7 days, the mice were inoculated intraperitoneally with 2.5 ml of 2.98% Difco Fluid Thioglycollate medium (BD, Franklin Lakes, NJ, USA), then fed for 3–4 days. The mice were then euthanized, and the peritoneal cavities were flushed twice with 10 ml of cold PBS. Cell suspensions were centrifuged at 1000×*g* for 10 min, washed twice in 5 ml of RPMI 1640 medium (Biological Industries) and diluted in RPMI 1640 medium with 10% FBS (Biological Industries) to a final concentration of 1.5 × 10^6^ cells/ml. The cells were transferred to 6-well cell culture plates (4.5 × 10^6^ cells/well) and allowed to settle for 4–6 h. The medium was replaced with fresh culture medium, and the cells were cultured for 12 h at 37 °C with 5% CO_2_. Cell purity was verified using flow cytometry detection of the CD11b marker (> 98%).

### Cell line culture and establishment of the stimulation model

The THP-1 human monocytic cell line (ATCC TIB-202; American Type Culture Collection) was cultured in RPMI-1640 medium supplemented with 10% FBS in a T75 flask and maintained at 37 °C and 5% CO_2_. The cells were transferred into 12-well cell culture plates (1.5 × 10^6^ cells/well) and induced to differentiate with 50 nM 4α-phorbol 12-myristate 13-acetate-PMA (Sigma-Aldrich) for 48 h. After washing three times with PBS, the THP-1 cells were cultured in complete RPMI-1640 medium for stimulation experiments.

Mouse macrophages or THP-1 cells were prestimulated with 25 μg/ml GEVs for 1 h and then infected with 1.5 × 10^6^ *G. duodenalis*/ml for 18 h in a noncontact system. An equal volume of a PBS-treated group was used as a negative control. The GEV (25 μg/mL)-treated group or a noncontact system for the *G. duodenalis* (1.5 × 10^6^ parasites/ml)-treated group was used as a positive control group, as described in our previous study [20]. Briefly, a 0.4-μm-sized Transwell insert (Corning Inc., Corning, NY, USA) was added to cell plates that were previously coated with cultured macrophages, and *G. duodenalis* was inoculated onto the membrane of the Transwell apparatus.

### Real-time quantitative PCR assays

Total RNA was extracted from infected mouse macrophages with TRIzol reagent (Monad; Wuhan, China) after 12 h of incubation with GEVs. Purity and concentration were verified (OD_260 nm_/OD_280 nm_ = 1.8–2.0) with a Nanodrop ND-2000 spectrophotometer (Thermo Fisher Scientific). First-strand cDNA was synthesized via reverse transcription with MonScript RTIII Super Mix with dsDNase (Two-Step) (Monad). Primers for proinflammatory cytokines and housekeeping genes were synthesized by Comate Bioscience Company Limited (Changchun, China) (see Table 1 for primer sequences). Gene transcription levels were detected using real-time quantitative PCR (qPCR) assays performed on a LightCycler 480 II platform (Roche Diagnostics GmbH, Mannheim, Germany) with the MonAmp SYBR Green qPCR Mix (None ROX; Monad). The assays were performed in a reaction mixture (total volume 20 μl) containing 10 μl of 2× qPCR Mix, 0.2 μM of forward primer, 0.2 μM of reverse primer, 1 μl of cDNA diluted 20-fold and nuclease-free water. The reaction program consisted of denaturation at 95 °C for 30 s; amplification at 95 °C/10 s, 60 °C/30 s for 40 cycles (the fluorescence signal was read at this point); a default melting curve was used. Gene expression analysis was normalized to the expression of Actb (beta-actin), and the relative mRNA fold-change was calculated by the 2^−ΔΔCq^ method, where ΔCq represents the Cq (proinflammatory cytokines) − Cq (Actb), and ΔΔCq represents the ΔCq (experimental group) − ΔCq (control group).

**Table 1:** Primer sequences used for the real-time quantittive PCR assays

Target	Genebank number	Primer sequences (5′ to 3′)	Product size (bp)	Primer length (nt)	Cross intron length (nt)	Primer site
IL-1β	NM_008361	F: AGGAGAACCAAGCAACGACA	241	20	1545	582…601
		R: CTCTGCTTGTGAGGTGCTGA		20		822…803
TNF-α	NM_013693	F: GACGTGGAACTGGCAGAAGA	253	20	696	192…211
		R: GGCTACAGGCTTGTCACTCG		20		446…427
IL-6	NC_000071	F: TGCCTTCTTGGGACTGATGC	216	20	1272	279…298
		R: GCAAGTGCATCATCGTTGTTC		21		1765…1745
Actb	NM_007393	F: GCCATGTACGTAGCCATCCA	240	20	455	391…410
		R: ACGCACGATTTCCCTCTCAG		20		630…611

*Actb* Beta-actin gene,* F* forward primer,* IL* interleukin, *R* reverse primer,* TNF-α* tumor necrosis factor alpha

### Enzyme-linked immunosorbent assays

Cell culture supernatants were collected 18 h after stimulation to detect secretion of the proinflammatory cytokines interleukin (IL)-1β, IL-6 and tumor necrosis factor alpha (TNF-α) with commercial enzyme-linked immunosorbent assays (ELISA) kits (Invitrogen, Thermo Fisher Scientific, Carlsbad, CA, USA). According to the manufacturer’s instructions, ELISA plates (Corning Inc.,) were coated with capture antibodies (100 μl/well) and incubated at 4 °C overnight. The plates were blocked with ELISA/ELISPOT diluent (1× 200 μl/well) at room temperature (RT) for 1 h after being washed three times with wash buffer. Prepared twofold serial dilutions of standard samples and supernatant samples (100 μl/well) were added to the plates and incubated overnight at 4 °C. After five washes, detection antibodies (100 μl/well) were added to the plates and incubated at RT for 1 h. Streptavidin-horseradish peroxidase (HRP) or avidin-HRP (100 μl/well) and a 1× TMB solution (100 μl/well) were added to plates and incubated for 30 min and 15 min, respectively. The reaction was stopped with the addition of a stop solution (100 μl/well), following which the cells were washed seven times. The OD_450 nm_ values were measured on a microplate reader, and standard curves were generated. The OD_450 nm_ values of the samples were converted to protein concentrations.

### Western blot assays

Cells were collected at 0, 0.5, 1, 2 and 4 h after GEV stimulation, and protein was extracted using RIPA lysis buffer (Solarbio, Beijing, China) supplemented with 100× protease inhibitor cocktail (1% v/v) and 10× phosphatase inhibitor complex III (10% v/v; Sangon, Shanghai, China). The lysis supernatants were quantified with a BCA protein assay kit. Protein samples were denatured by mixing with 6× protein loading buffer (TransGen, Beijing, China) and boiling for 10 min. Equal amounts of protein samples (20 μg/well) were added to a 12% sodium dodecyl sulfate-polyacrylamide (SDS-PAGE) gel and separated by electrophoresis (80 V/1 h and 120 V/1 h) in Tris–glycine–SDS buffer. The protein samples were transferred to polyvinyl difluoride membranes (0.45 μm, Merck Millipore) under conditions of 200 mA/1.5 h, and the membranes were blocked in 5% skim milk (in TBST buffer) at RT for 2 h and incubated overnight with primary antibodies against p38 MAPK, phospho-p38 MAPK, p44/42 MAPK (Erk1/2), phospho-p44/42 MAPK (Erk1/2), AKT, phospho-AKT, IKKα, IKKβ, phospho-IKKα/β, NF-κB p65, phospho-NF-κB p65, IκBα, phospho-IκBα (1:1000; Cell Signaling Technology Inc., Danvers, MA, USA) and β-actin (1:5000; Proteintech, Wuhan, China) at 4 °C overnight. Membranes were washed three times with TBST (5 min/wash) and then incubated with HRP-conjugated goat anti-rabbit immunoglobulin G (IgG) or horse anti-mouse IgG secondary antibody (1:1000, Cell Signaling Technology Inc.) at RT for 1 h. The membranes were washed again with TBST and developed using an Omni-ECL Pico light chemiluminescence kit (Epizyme, Shanghai, China). The blots were visualized on a ChemiScope western blot imaging system (Clinx, Shanghai, China) and analyzed using ImageJ software.

### Inhibition assays

To examine the roles of activated pathways in response to the GEV-induced proinflammatory immune reaction in mouse macrophages, cells were pretreated with an inhibitor of p38 MAPK (SB203580, 30 μM; MedChemExpress, USA), an inhibitor of ERK (SCH772984, 300 nM; MedChemExpress LLC, Monmouth Junction, NJ, USA), an inhibitor of AKT1/2/3 (MK-2206 2HCl, 5 μM; Selleck, Shanghai, China) or an inhibitor of IκBα phosphorylation (BAY 11-7082, 5 μM; Selleck) for 2 h before stimulation. Unpretreated cells were used as controls. The mRNA fold-change of the activated inflammatory cytokines was determined using qPCR, and protein expression levels in the supernatants and cells were determined using ELISAs and western blotting assays, respectively.

### Immunofluorescence assays

To observe the subcellular localization of NF-κB p65, 5 × 10^5^ mouse macrophages previously coated onto 24-well plates were stimulated with 25 μg/ml GEVs for 0 or 1 h. The cells were gently washed three times with warm TBS, fixed in 4% paraformaldehyde (Biosharp, Beijing, China) for 10 min at RT, washed three times with TBS and permeabilized in 0.1% Triton X-100 at RT for 20 min. The infected cells were blocked in 5% BSA (in TBS) at RT for 2 h, incubated overnight with rabbit anti-phospho-NF-κB p65 antibodies (1:100) at 4 °C, washed three times in TBST and incubated with FITC AffiniPure goat anti-rabbit IgG (H + L) (1:100; EarthOx Life Sciences, Millbrae, CA, USA) antibodies in the dark for 1 h at 37 °C. After washing, cell nuclei were stained with 4′,6-diamidino-2-phenylindole and dihydrochloride (DAPI, 1 μg/mL; Thermo Fisher Scientific). The subcellular localization of NF-κB p65 was observed under a fluorescence microscope (Olympus, Tokyo, Japan).

### Statistical analysis

The results are presented as the mean ± standard error of the mean (SEM) of three biological replicates. Differences between two groups were analyzed using the *t* test with GraphPad Prism version 7.00 software (GraphPad Software Inc., La Jolla, CA, USA), and multiple groups were analyzed by one-way analysis of variance using SPSS version 22.0 software (SPSS-IBM Corp., Armonk, NY, USA). The homogeneity of variance of the data was analyzed with the Levene test, followed by a Bonferroni post hoc test (B). Graphs were generated in GraphPad Prism 7.00. Significance is shown as *p* < 0.05, *p* < 0.01 and *p* < 0.001, and n.s. indicates “not significant” (*p* > 0.05).

## Results

### GEVs enhanced *G. duodenalis-*induced proinflammatory cytokine production in macrophages

**Fig. 1:**
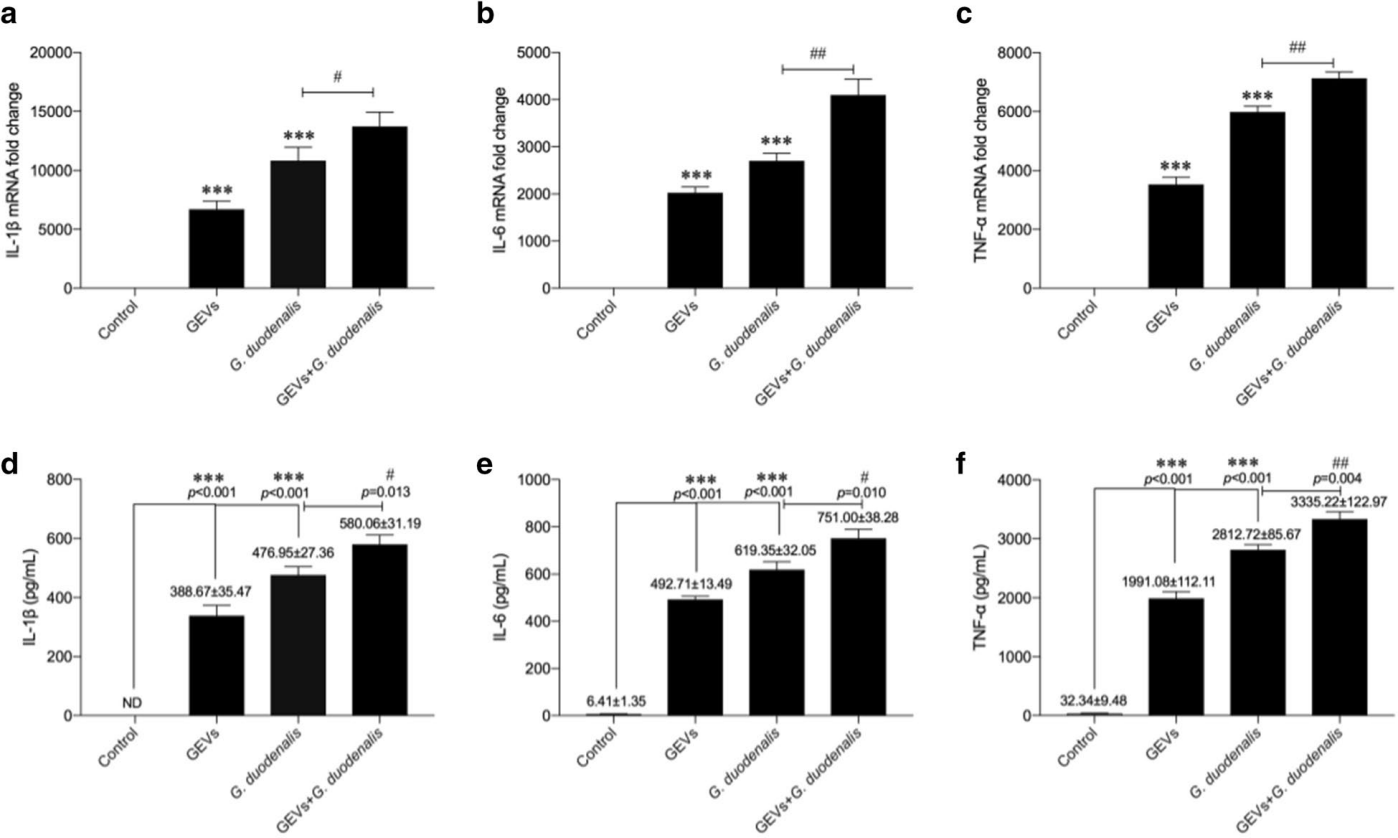
Treatment with *Giardia duodenalis* extracellular vesicles (*GEVs*) enhanced proinflammatory cytokine transcription and secretion from mouse macrophages. Cells were inoculated with 25 μg/ml GEVs, 1.5 × 10^6^ *G. duodenalis*/ml, or GEVs + *G. duodenalis*. **a–c** Real-time quantitative PCR analysis of the transcription levels of the proinflammatory cytokines interleukin* (IL*)-1β, IL-6 and tumor necrosis factor alpha (*TNF-α*) in infected cells collected at 12 h. **d–f** Measurement of the secretion levels of proinflammatory cytokines in the supernatants collected 18 h after inoculation, by enzyme-linked immunosorbent assay (ELISA). The results are shown as the mean ± standard error of the mean (SEM) of triplicate experiments. Asterisks indicate significance level of difference *vs *the phosphate buffered saline control: *p < 0.05, **p < 0.01, ***p < 0.001. Hashtag symbols indicate significance level of difference* vs* *G. duodenalis*-treated control: ^#^*p* < 0.05, ^##^*p* < 0.01

A previous study showed that *G. duodenalis* increased proinflammatory cytokine production in mouse macrophages [28]. To examine the role of GEVs in *G. duodenalis-*induced proinflammatory cytokine production, cells were pretreated with GEVs for 1 h, then inoculated with *G. duodenalis* for measurement of proinflammatory cytokine expression. The negative and positive control groups were those treated with PBS + GEVs and PBS + *G. duodenalis*, respectively. The mRNA levels of proinflammatory cytokines were measured by qPCR (Fig. 1a–c), and the protein secretion levels were measured with ELISAs (Fig. 1d–f). The results showed that both the GEV and *G. duodenalis* treatment groups exhibited significantly upregulated proinflammatory cytokine transcription and expression compared to the PBS-negative control group (***p < 0.001). GEVs enhanced the *G. duodenalis-*induced inflammatory response of murine macrophages in the GEVs + *G. duodenalis* group compared to the *G. duodenalis* single-treatment group (*p < 0.05 or **p < 0.01). A similar trend was obtained with the stimulated THP-1 cells (Additional file 1: Fig. S1). Overall, these data demonstrate that GEVs induce proinflammatory cytokine production in macrophages and that GEVs can enhance the *G. duodenalis-*induced inflammatory response.

### GEVs activated the p38, ERK and AKT signaling pathways in mouse macrophages

**Fig. 2:**
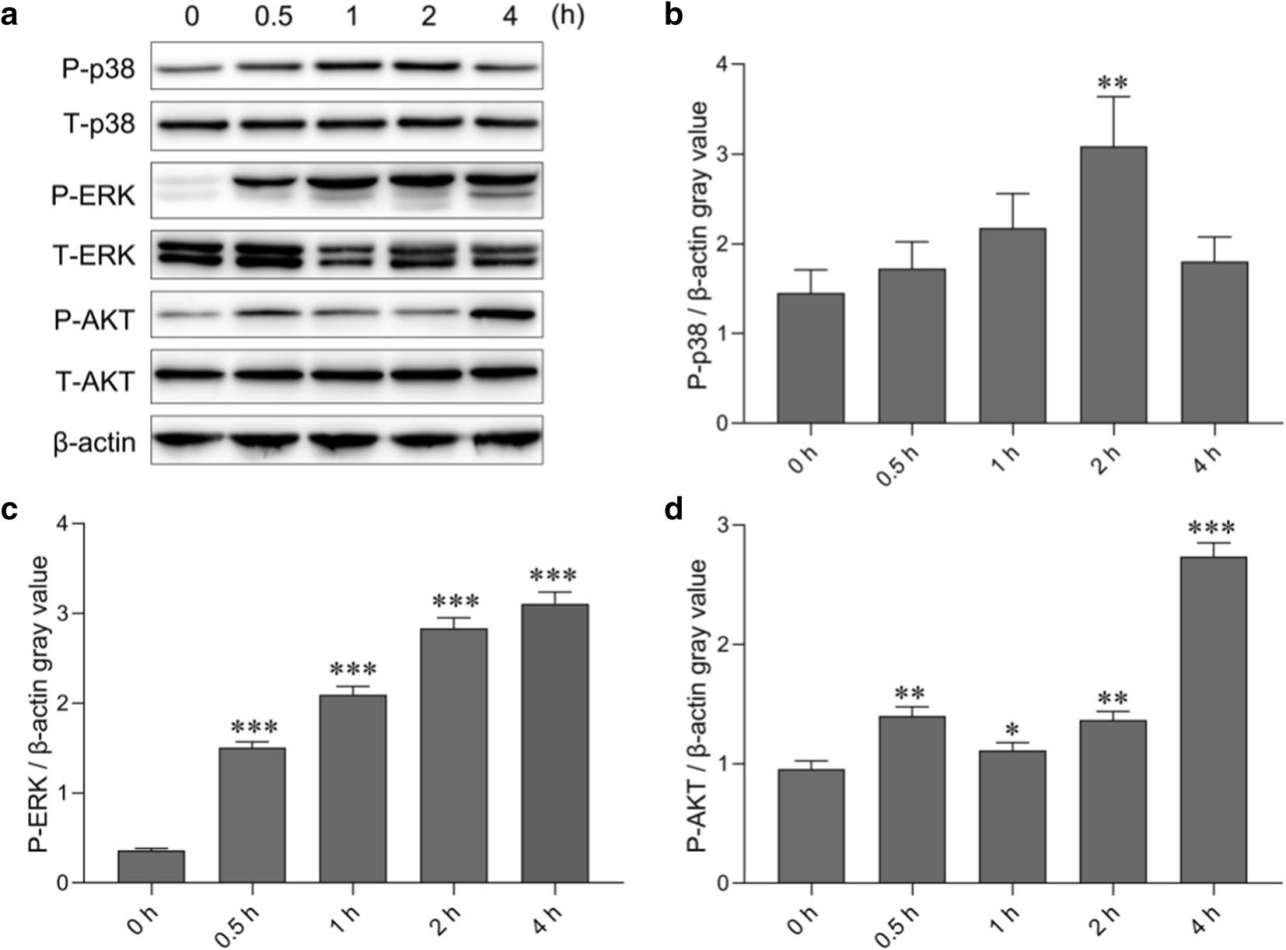
p38 MAPK, ERK and Akt signaling pathway activation in response to GEV stimulation of mouse macrophages. **a** GEVs were inoculated into mouse macrophages, and cells were collected at the indicated time points for measurement of total protein and phosphorylated protein expression using western blotting. **b** Gray value analysis of phospho-p38/β-actin. **c** Gray value analysis of phospho-ERK/β-actin. **d** Gray value analysis of phospho-AKT/β-actin. The results are shown as the mean ± SEM of triplicate experiments. Significant differences* vs* 0-h control: *p < 0.05, **p < 0.01, ***p < 0.001

The MAPK and AKT signaling pathways are involved in different pathogen-triggered immune responses. To examine the roles of these signaling pathways in GEV-induced proinflammatory cytokine production, GEVs were inoculated into mouse macrophages, and the cells were collected at 0, 0.5, 1, 2 and 4 h after inoculation to measure the levels of total protein and phosphorylated protein in the MAPK and AKT signaling pathways using western blot assays. As shown in Fig. 2a, GEVs induced phosphorylation of p38, ERK and AKT. Protein gray values of phospho-p38/β-actin, phospho-ERK/β-actin and phospho-AKT/β-actin were separately calculated using Excel (Microsoft Corp., Redmond, WA, USA) software, and differences were analyzed using SPSS software. The results showed that compared to the phospho-p38 levels at 0 h, the p38 phosphorylation levels began to increase between 0.5 and 1 h (*p* > 0.05), peaked at 2 h (*p* < 0.01) after GEV inoculation and began to decrease at 4 h. However, the levels remained higher at 4 h compared to those at 0 h (*p* > 0.05; Fig. 2b). The ERK phosphorylation levels increased with increasing inoculation time and peaked at 4 h compared to those at 0 h (*p* < 0.001; Fig. 2c). The AKT phosphorylation levels slowly increased between 0.5 and 2 h (*p* < 0.05 or *p* < 0.01) and reached their highest levels at 4 h (*p* < 0.001; Fig. 2d). These results indicated that the p38, ERK and AKT signaling pathways were activated after the inoculation of GEVs into mouse macrophages.

### GEVs enhanced proinflammatory cytokine production through phosphorylation of p38 and ERK

**Fig. 3:**
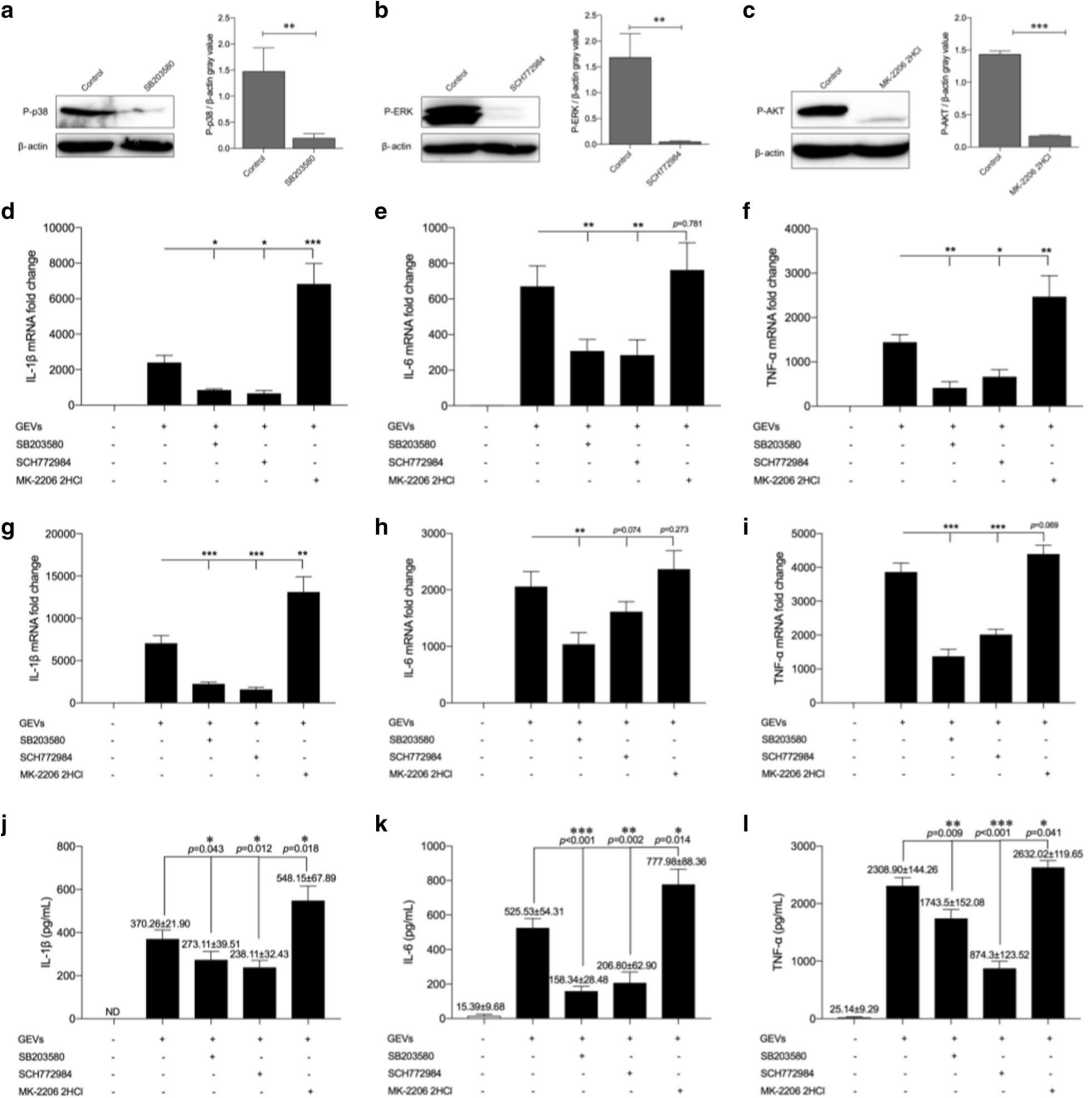
GEVs regulated proinflammatory cytokine transcription and secretion through the p38 MAPK and ERK signaling pathways in a positive feedback manner and the Akt signaling pathway in a negative feedback manner. Mouse macrophages were pretreated with the p38 MAPK inhibitor SB203580 (30 μM), ERK inhibitor SCH772984 (300 nM) or AKT inhibitor MK-2206 2HCl (5 μM) for 2 h. Unpretreated groups were used as controls. The inhibited cell groups were inoculated with GEVs for 2, 4 and 4 h. **a–c** The phosphorylated protein expression levels of p38, ERK and AKT were measured using western blotting, and the gray values of phosphorylated protein/β-actin were calculated using ImageJ software. **d–f** Levels of proinflammatory cytokine transcription levels in cells collected 6 h after inoculation were detected using real-time quantitative PCR assays (qPCR). **g–i** Levels of proinflammatory cytokine transcription levels in cells collected 12 h after inoculation were detected using qPCR assays. **j–l** Levels of proinflammatory cytokine protein secretion levels were detected in supernatants collected 18 h after inoculation using ELISAs. The results show the mean ± SEM of triplicate experiments. Signficant differences* vs* noninhibitor treatment control: *p < 0.05, **p < 0.01, ***p < 0.001

To examine the roles of the activated p38 and ERK signaling pathways in GEV-induced proinflammatory cytokine production, inhibitors of p38 MAPK-SB203580 and ERK-SCH772984 were added separately to mouse macrophages, followed by inoculation of GEVs after pretreatment with inhibitors for 2 h. At 2 or 4 h after inhibitor treatment, the cells were collected for separate measurement of the protein levels of phospho-p38 and phospho-ERK. Cells or cell culture supernatants were collected 12 and 18 h after treatment, respectively, for measurement of inflammatory cytokine expression. As shown in Fig. 3a, b, the phosphorylation levels of p38 and ERK were successfully inhibited at 2 and 4 h, respectively. The mRNA levels of inflammatory cytokines were significantly downregulated after pretreatment with SB203580 or SCH772984 (*p* < 0.05, *p* < 0.01 or *p* < 0.001) except for IL-6 at 12 h, which was slightly reduced (*p* > 0.05) after pretreatment with SCH772984 compared with that in the “no inhibitor” pretreatment groups (Fig. 3d, e, g, h). The ELISA results showed that pretreatment with SB203580 or SCH772984 slightly decreased IL-1β secretion (*p* < 0.05) and dramatically reduced the secretion of IL-6 and TNF-α (*p* < 0.01 and *p* < 0.001, respectively) (Fig. 3j–l). Inhibition assays revealed that GEVs regulated proinflammatory cytokine production through phosphorylation of p38 and ERK in a positive feedback manner.

### GEVs inhibited proinflammatory cytokine production through phosphorylation of AKT

To examine the role of the activated AKT signaling pathway in the GEV-induced inflammatory response, cells were pretreated with the AKT inhibitor MK-2206 2HCl for 2 h then stimulated with GEVs. The protein level of phospho-AKT in the infected cells was determined 4 h after GEV treatment using western blotting, and mRNA levels of inflammatory cytokines in infected cells were measured using qPCR 12 h after GEV treatment. The protein levels of inflammatory cytokines from infected cells were detected via ELISAs 18 h after GEV treatment. The levels of AKT phosphorylation were significantly downregulated in the MK-2206 2HCl pretreatment group compared with those in the untreated group (*p* < 0.001; Fig. 3c). The mRNA levels of IL-1β mRNA were dramatically increased compared with that in the untreated group (*p* < 0.01 or *p* < 0.001). The levels of IL-6 and TNF-α were upregulated, albeit not significantly (*p* > 0.05) except for TNF-α at 6 h, which was markedly enhanced compared to the “no inhibitor” pretreatment group (Fig. 3d–f, g–i). The results of the ELISAs for proinflammatory cytokine secretion showed a significantly increasing trend after cell pretreatment with MK-2206 2HCl (*p* < 0.05; Fig. 3j–l). These results demonstrated that GEVs regulated proinflammatory cytokine production via the phosphorylation of AKT in a negative feedback manner.

### GEVs activated NF-κB signaling pathways in mouse macrophages

**Fig. 4:**
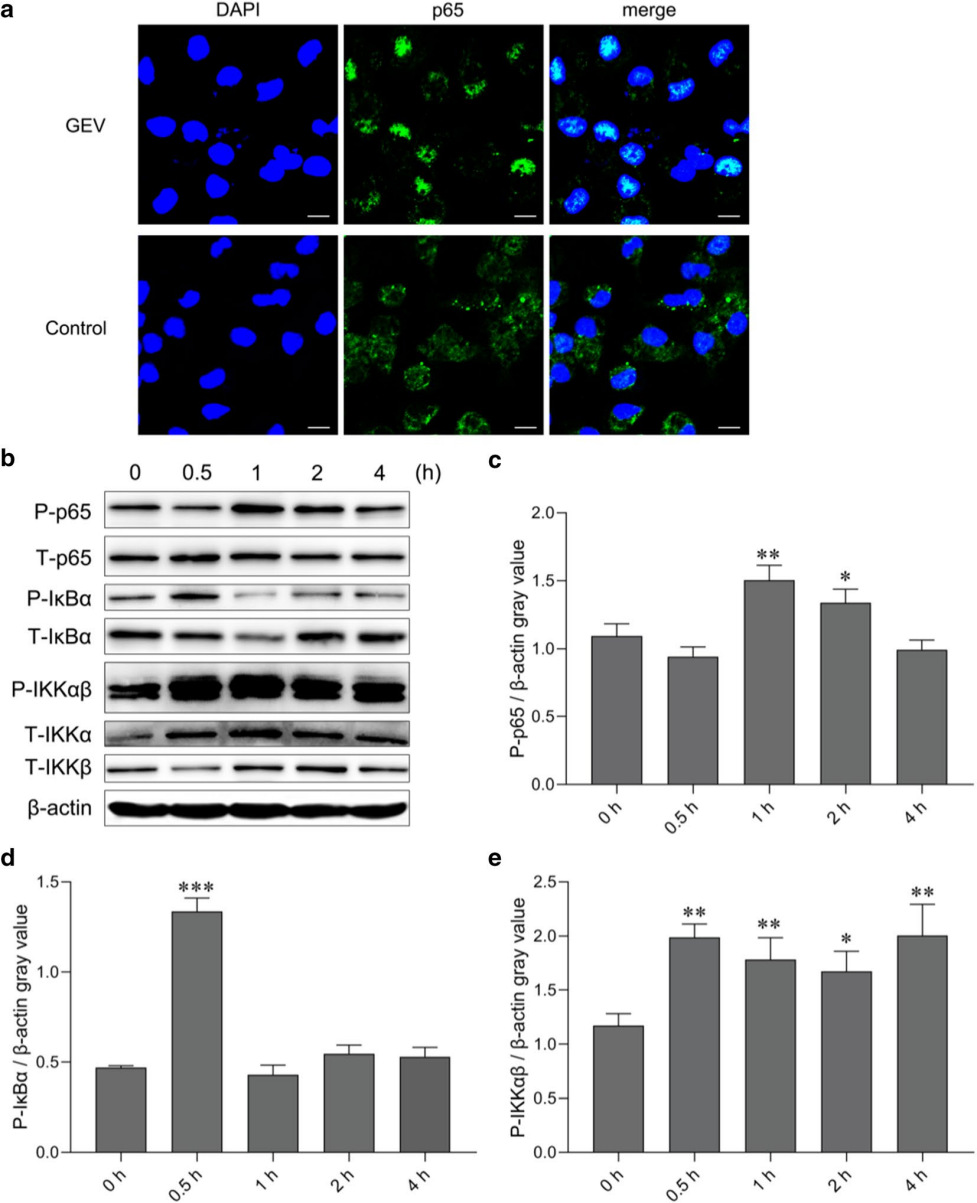
NF-κB signaling pathway activation in response to GEV stimulation in mouse macrophages. **a** GEVs were inoculated into mouse macrophages previously coated onto coverslips for 1 h, and immunofluorescence staining analysis of the subcellular location of NF-κB p65 was performed. Scale bars: 10 μm. **b** GEVs were inoculated into mouse macrophages, and cells were collected at the indicated time points for measurement of total protein and phosphorylated NF-κB p65, IκBα, IKKα and IKKβ protein expression levels by western blotting. **c** Gray value analysis of phospho-p65/β-actin. **d** Gray value analysis of phospho-IκBα/β-actin. **e** Gray value analysis of phospho-IKKαβ/β-actin. The results are shown as the mean ± SEM of triplicate experiments. Significant differences* vs* 0-h control: *p < 0.05, **p < 0.01, ***p < 0.001

NF-κB is a nuclear transcription factor that regulates the expression of proinflammatory cytokines. To determine whether the NF-κB signaling pathway was activated after the inoculation of mouse macrophages with GEVs, cells were collected 0, 0.5, 1, 2 and 4 h after GEV inoculation for the measurement of total and phosphorylated protein levels of p65, IKKα, IKKβ and IκBα by western blot assays, and the location of NF-κB p65 was observed with immunofluorescence staining 1 h after inoculation with GEVs. The localization results showed that NF-κB p65 primarily existed in the cytoplasm of the untreated cells and that most NF-κB p65 was transferred to the cell nucleus and colocalized with the nucleus 1 h after mouse macrophage inoculation with GEVs (Fig. 4a). The phosphorylation levels of NF-κB p65, IκBα and IKKαβ in the infected cells varied during the initial 4 h of monitoring (Fig. 4b). The gray values of phospho-p65/β-actin remained unchanged at 0.5 h (*p* > 0.05), began to increase between 1 and 2 h (*p* < 0.05 or *p* < 0.01), then decreased at 4 h (*p* > 0.05; Fig. 4c). The gray values of phospho-IκBα/β-actin dramatically increased during the initial stimulation stage, as measured at 0.5 h (*p* < 0.001), then returned to normal expression levels between 1 and 4 h (*p* > 0.05; Fig. 4d). The gray values of phospho-IKKαβ/β-actin markedly increased to high levels at 0.5 h and lasted for 4 h (*p* < 0.05 or *p* < 0.01; Fig. 4e). These data demonstrated that the NF-κB signaling pathway was activated after mouse macrophage inoculation with GEVs.

### GEVs enhanced proinflammatory cytokine production via phosphorylation of IκBα

**Fig. 5:**
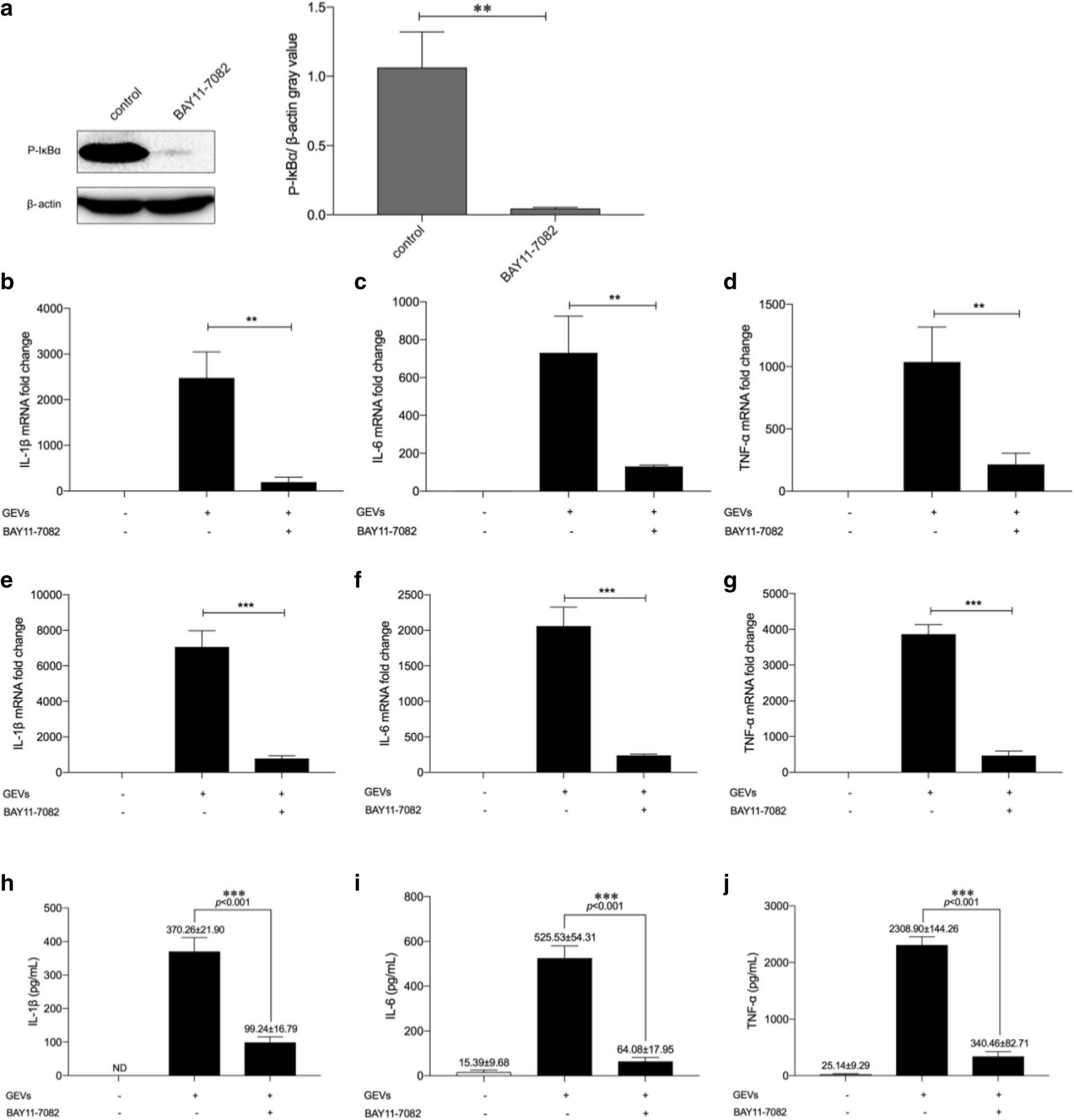
GEVs regulated proinflammatory cytokine transcription and secretion through NF-κB signaling pathways in a positive feedback manner. Mouse macrophages were pretreated with the IκBα phosphorylation inhibitor BAY 11-7082 (5 μM) for 2 h. Groups not pretreated were used as controls. The cells were inoculated with GEVs for 0.5 h. **a** The protein expression levels of phosphorylated phospho-IκBα were measured by western blotting, and gray values of phosphorylated protein/β-actin were calculated using ImageJ software. **b–d** Transcription levels of proinflammatory cytokines in cells collected 6 h after inoculation were determined using qPCR assays. **e–g** Transcription levels of proinflammatory cytokines in cells collected 12 h after inoculation were determined using qPCR assays. **h–j** The proinflammatory cytokine protein secretion levels in the supernatants collected 18 h after inoculation were detected using ELISAs. The results are shown as the mean ± SEM of triplicate experiments. Significant differences* vs* noninhibitor treatment control: **p < 0.01 , ***p < 0.001.* ND *Not determined

The translocation of NF-κB p65 was primarily due to the degradation of total IκBα. To examine the influence of NF-κB on the GEV-induced inflammatory response, an inhibitor of IκBα phosphorylation, BAY 11-7082, was used to inhibit IκBα degradation. The western blot assay results showed that the expression levels of phospho-IκBα were almost completely inhibited (*p* < 0.01; Fig. 5a). The mRNA and secretion levels of inflammatory cytokines were measured using qPCR and ELISAs, respectively. As shown in Fig. 5b–j, the inflammatory response was significantly inhibited compared with that in infected cells without BAY 11-7082 pretreatment (*p* < 0.01 or *p* < 0.001). Overall, these results indicated that the NF-κB signaling pathway regulated the GEV-induced inflammatory response in a positive feedback manner.

## Discussion

Increasingly more attention is focusing on *G. duodenalis-*triggered diarrheal diseases, especially in developing countries, also driven by the increasing resistance to anti-giardiasis drugs [29]. The innate immune system is involved in the development of various diseases through its recognition of PAMPs from pathogens. EVs carry pathogen-derived PAMPs to communicate with host cells and are involved in the innate immunity response [30]. Therefore, an in-depth study of the role of GEVs in the invasion of *G. duodenalis* into host cells and the underlying immune response mechanisms should contribute to a better knowledge of *G. intestinalis*–host interactions. Macrophages are the most important member of the innate immune system and play a vital role in resisting the invasion of multiple pathogens. In the present study, we examined the role of GEVs in the *G. duodenalis*-induced inflammatory response using mouse macrophages as models and determined the mechanisms by which GEVs trigger the inflammatory immune response.

Eukaryotic cells secrete EVs, which carry a cargo of biological molecules that mediate communication between cells [31]. *Staphylococcus aureus*-derived EVs increase the secretion of proinflammatory cytokines and T helper 2-biasing mediators, such as thymic stromal lymphopoietin, M1 inflammatory protein-1a and eotaxin production, all of which increase the number of eosinophils and enhance disease development [32]. Highly adherent strains of *Trichomonas vaginalis* release EVs that augment attachment to human cervical epithelial cells of poorly adherent strains [14]. Studies on *G. duodenalis* performed to date have generally focused on the role of EVs in the attachment of the parasite to intestinal epithelial cells, and few studies have examined the regulatory mechanisms of GEV-induced immune responses in hosts. Evans-Osses et al. reported that *G. duodenalis* microvesicles increased the activation and allostimulation of human dendritic cells [16, 17], and our recently published study showed that GEVs regulate host cell innate immunity via the TLR2 and NLRP3 inflammasome signaling pathways [20]. In the present study, we inoculated GEVs, *G. duodenalis* and GEVs + *G. duodenalis* into mouse macrophages and then measured the secretion levels of the proinflammatory cytokines IL-1β, IL-6 and TNF-α. The results showed that both GEVs and *G. duodenalis* alone were able to trigger the production of these three proinflammatory cytokines and that GEV pretreatment enhanced the *G. duodenalis*-induced inflammatory response in mouse macrophages.

A previous study showed that inflammasome-derived IL-1β secretion induced IL-1R/MyD88 signaling to trigger nitric oxide production, which enabled the killing of parasites, such as *Leishmania*, *Plasmodium* and *Toxoplasma gondii* [33]. IL-6 and TNF-α share of the ability to clear *G.* *duodenalis* in mouse infection models [34, 35]. The enhanced levels of IL-1β, IL-6 and TNF-α triggered by GEVs may contribute to host resistance to disease development.

The MAPK signaling pathway regulates cell growth and differentiation and controls cellular responses to cytokines and stress [22]. The PI3K/Akt signaling pathway participates in cell survival or apoptosis [26]. The NF-κB signaling pathway is involved in the biological processes of immunity, inflammation and stress response [24]. Previous studies showed that extracellular products secreted by *G. lamblia* GS induced IL-8 secretion in HT-29 cells through activation of the p38, ERK and NF-κB pathways [36]. Mice deficient in TLR2 showed attenuation of giardiasis by increasing proinflammatory cytokine secretion, which was dependent on the AKT signaling pathway. In our present study, GEVs were inoculated into mouse macrophages, and total protein and phosphorylated protein levels of the MAPK, Akt and NF-κB signaling pathways were measured in infected cells 0, 0.5, 1, 2 and 4 h after inoculation. The results showed that GEVs phosphorylated p38 and ERK proteins, and inhibitory assays indicated that this process promoted proinflammatory cytokine secretion. GEVs also activated the AKT signaling pathway, and inhibition of AKT phosphorylation enhanced proinflammatory cytokine production. The inhibitory functions of AKT with MAPK were consistent with previously reported results that showed AKT negative feedback control of IL-12 production in dendritic cells [37]. NF-κB p65 is inhibited upon binding to IκB and remains inactive in the cytoplasm in classical signaling pathways. Stimulation with lipopolysaccharides, growth factors or antigen receptors activates the IKK complex (i.e. IKKβ, IKKα and NEMO), which phosphorylates IκB. Phosphorylation of IκB triggers self-ubiquitination and degradation to cause the release of p65. After posttranslational modification, such as phosphorylation, acetylation or glycation, activated p65 is transported into the cell nucleus and induces gene expression, either alone or in combination with the transcription factors AP-1, ETs or Stat [24, 38]. GEVs induced activation of the classical NF-κB signaling pathway through phosphorylation of IκBα, IKKα and IKKβ and translocation of NF-κB p65 into the nucleus in our study. Inhibition of IκBα phosphorylation decreased GEV-induced inflammatory response levels in mouse macrophages.

## Conclusions

The present study demonstrated that GEVs enhanced *G.* *duodenalis-*induced inflammatory response levels in mouse macrophages. Activation of the p38, ERK and NF-κB signaling pathways facilitated proinflammatory cytokine secretion and activation of the AKT signaling pathway to reduce proinflammatory cytokine production. The extensive research into the role of GEVs in regulating host cell inflammatory responses* in vitro* presented in this study provides data for exploring the underlying mechanisms in *G. duodenalis*–host interactions.

## Supplementary Information

**Additional file 1: Figure S1.** GEVs enhanced proinflammatory cytokines secretion from THP-1 cells. THP-1 cells were inoculated with 25 μg/ml GEVs, 1.5 × 106* G. duodenalis*/ml, or GEVs combined with* G. duodenalis* (GEVS +* G. duodenalis*). ELISA measurements of the secretion levels of proinflammatory cytokines IL-1β (**a**), IL-6 (**b**) and TNF-α (**c**) in the supernatants collected 18 h after inoculation. The results are presented as the mean ± standard error of the mean of triplicate experiments. Asterisks indicate significance level of difference *vs *the phosphate buffered saline control: *p < 0.05, **p < 0.01, ***p < 0.001. Hashtag symbols indicate significance level of difference* vs* *G. duodenalis*-treated control: ^#^*p* < 0.05, ^##^ *p* < 0.01.* n.s.* Not significant (*p* > 0.05).

## Data Availability

The data supporting the conclusions of this article are provided within the article. The original datasets analyzed in the present study are available from the corresponding author upon request.
