# Supplementary material for: Giardia duodenalis extracellular vesicles regulate the proinflammatory immune response in mouse macrophages in vitro via the MAPK, AKT and NF-κB pathways
Source: Parasit Vectors. 2021 Jul 8;14:358. doi: 10.1186/s13071-021-04865-5 (PMC8268305; doi:10.1186/s13071-021-04865-5)

**Additional figure captions**

**Fig. S1.** GEVs enhanced proinflammatory cytokines secretion from THP-1 cells. THP-1 cells were inoculated with 25 μg/mL GEVs, 1.5 × 10^6^ *G. duodenalis*/mL, or GEVs combined with *G. duodenalis*. ELISA measurements of the secretion levels of proinflammatory cytokines IL-1β (**a**), IL-6 (**b**) and TNF-α (**c**) in the supernatants collected 18 h after inoculation. The results are the mean±SEM of triplicate experiments. **p* < 0.05 or ****p* <0.001 vs. PBS-treated control. ^#^*p* < 0.05 or ^##^*p* < 0.01 vs. *G. duodenalis*-treated control. n.s. indicates not significant (*p* >0.05).

**Fig. S1**


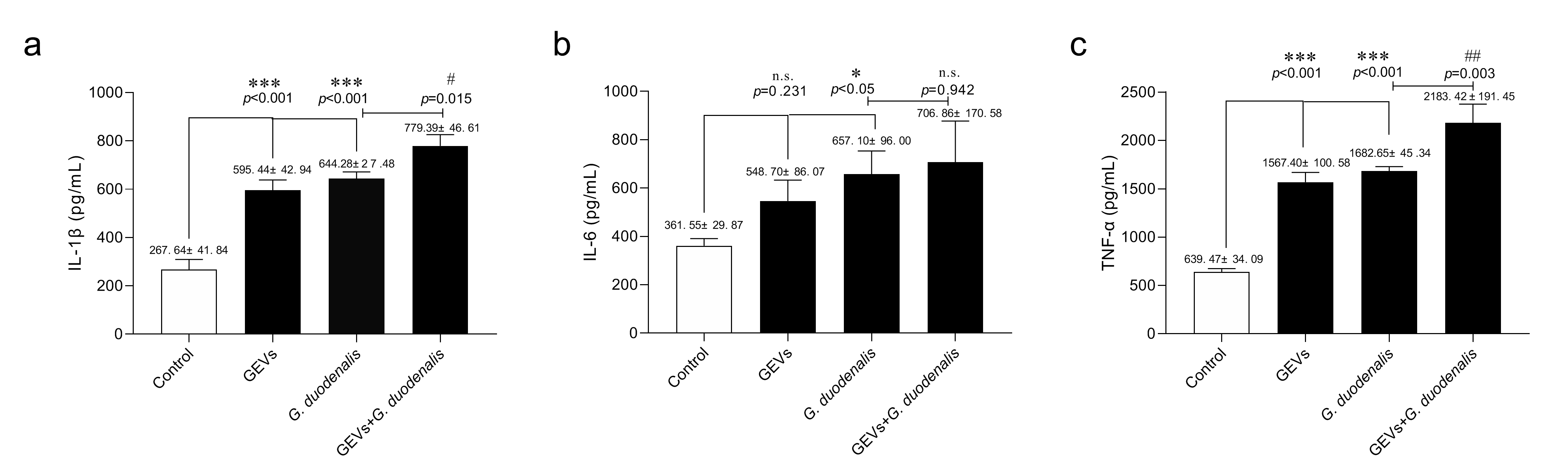

Supplement: Supplementary file 1 — Additional file 1: Figure S1. GEVs enhanced proinflammatory cytokines secretion from THP-1 cells. THP-1 cells were inoculated with 25 μg/ml GEVs, 1.5 × 10^6^ G. duodenalis/ml, or GEVs combined with G. duodenalis (GEVS + G. duodenalis). ELISA measurements of the secretion levels of proinflammatory cytokines IL-1β (a), IL-6 (b) and TNF-α (c) in the supernatants collected 18 h after inoculation. The results are presented as the mean ± standard error of the mean of triplicate experiments. Asterisks indicate significance level of difference vs the phosphate buffered saline control: *p < 0.05, **p < 0.01, ***p < 0.001. Hashtag symbols indicate significance level of difference vs G. duodenalis-treated control: #p < 0.05, ## p < 0.01. n.s. Not significant (p > 0.05). [file 13071_2021_4865_MOESM1_ESM.docx]
